# Differential transcriptomic alterations in nasal versus lung tissue of acrolein-exposed rats

**DOI:** 10.3389/ftox.2023.1280230

**Published:** 2023-11-27

**Authors:** Devin I. Alewel, Thomas W. Jackson, Katherine M. Rentschler, Mette C. Schladweiler, Anna Astriab-Fisher, Stephen H. Gavett, Paul A. Evansky, Urmila P. Kodavanti

**Affiliations:** ^1^ Oak Ridge Institute for Science and Education Research Participation Program, U.S. Environmental Protection Agency, Durham, NC, United States; ^2^ Public Health and Integrated Toxicology Division, Center for Public Health and Environmental Assessment, U.S. Environmental Protection Agency, Durham, NC, United States

**Keywords:** acrolein, air pollution, nose, lung, glucocorticoids, cytokines, transcriptomics

## Abstract

**Introduction:** Acrolein is a significant component of anthropogenic and wildfire emissions, as well as cigarette smoke. Although acrolein primarily deposits in the upper respiratory tract upon inhalation, patterns of site-specific injury in nasal *versus* pulmonary tissues are not well characterized. This assessment is critical in the design of *in vitro* and *in vivo* studies performed for assessing health risk of irritant air pollutants.

**Methods:** In this study, male and female Wistar-Kyoto rats were exposed nose-only to air or acrolein. Rats in the acrolein exposure group were exposed to incremental concentrations of acrolein (0, 0.1, 0.316, 1 ppm) for the first 30 min, followed by a 3.5 h exposure at 3.16 ppm. In the first cohort of male and female rats, nasal and bronchoalveolar lavage fluids were analyzed for markers of inflammation, and in a second cohort of males, nasal airway and left lung tissues were used for mRNA sequencing.

**Results:** Protein leakage in nasal airways of acrolein-exposed rats was similar in both sexes; however, inflammatory cells and cytokine increases were more pronounced in males when compared to females. No consistent changes were noted in bronchoalveolar lavage fluid of males or females except for increases in total cells and IL-6. Acrolein-exposed male rats had 452 differentially expressed genes (DEGs) in nasal tissue *versus* only 95 in the lung. Pathway analysis of DEGs in the nose indicated acute phase response signaling, Nrf2-mediated oxidative stress, unfolded protein response, and other inflammatory pathways, whereas in the lung, xenobiotic metabolism pathways were changed. Genes associated with glucocorticoid and GPCR signaling were also changed in the nose but not in the lung.

**Discussion:** These data provide insights into inhaled acrolein-mediated sex-specific injury/inflammation in the nasal and pulmonary airways. The transcriptional response in the nose reflects acrolein-induced acute oxidative and cytokine signaling changes, which might have implications for upper airway inflammatory disease susceptibility.

## 1 Introduction

Acrolein, an electrophilic α,β-unsaturated aldehyde, is a volatile respiratory irritant formed during biomass burning, ambient photochemical transformation of anthropogenic pollutants, and tobacco combustion or pyrolysis (ATSDR, 2007; Stevens and Maier, 2008). The U.S. Environmental Protection Agency ranks acrolein as a prominent nationwide risk to health among hazardous air pollutants, and an estimated 97% of the non-carcinogenic respiratory hazard associated with mainstream cigarette smoke exposure is due to acrolein inhalation (U.S. EPA, 2006; Fowles and Dybing, 2003; Alwis et al., 2015). Acrolein exposure is implicated in the pathogenesis of multiple respiratory diseases, such as chronic obstructive pulmonary disease and lung cancer, and induces injury via sustained oxidative and inflammatory signaling, as well as tissue remodeling through extracellular matrix destruction and necrosis (Yeager et al., 2016).

In nose-breathing rodents, chemical reactivity with airway lining affects site-specific deposition and injury (Cichocki et al., 2014). Water-soluble vapors such as acrolein are scrubbed within the nasal turbinates, resulting in the stimulation of nasal mucosal trigeminal nerve endings (Alarie, 1973; Harkema, 1990). Chemo-sensory responses are largely mediated through nociceptive unmyelinated C-fibers, which are the most numerous neural projections in the airway (Mazzone and Undem, 2016; Gambeta et al., 2020). Acrolein activates Transient Receptor Potential Vanilloid and Ankyrin receptors (TRPV1 and TRPA1) at nerve endings, stimulating afferent signaling to the trigeminal ganglion and then the brain (Bautista et al., 2006; Achanta and Jordt, 2017). Some volatile gaseous pollutants with low solubility, such as ozone, deposit into the distal lung space, triggering vagal sensory neurons (Mazzone and Undem, 2016) to send nerve signals that traverse through the jugular and nodose ganglia and into the brain stem and stress-regulating regions such as the hypothalamus (Mazzone and Undem, 2016). Upper or lower airway chemical deposition following acrolein or ozone exposure triggers neurally mediated sympathetic-adrenal-medullary (SAM) and hypothalamic-pituitary-adrenal (HPA) neuroendocrine changes associated with hyperglycemia, circulating pituitary hormone alterations, and adrenal-hormone release (Miller et al., 2015; 2016; Snow et al., 2017; Henriquez et al., 2019; Alewel et al., 2023b).

Acute acrolein inhalation induces immediate bronchoconstriction (reduced ventilatory capacity), cardiovascular dysfunction, and release of adrenal catecholamines and glucocorticoids, which is believed to be modulated through a combined effort of sympathetic and parasympathetic innervation (Hazari et al., 2014; Snow et al., 2017). Following inhalation, acrolein rapidly reacts with cellular macromolecules to induce a myriad of airway epithelial effects, including depletion of airway antioxidant stores [e.g., glutathione (GSH)], production of reactive oxygen species, and induction of inflammatory cascades such as NFκB (Moghe et al., 2015). Acrolein also targets organelle-specific functions within the endoplasmic reticulum (ER) and mitochondria, resulting in protein unfolding and mitophagy (Moghe et al., 2015), which are etiological processes of chronic respiratory diseases associated with repeated acrolein exposure (Cloonan and Choi, 2016). Although the molecular effects of acrolein are fairly well characterized, a comparative assessment of transcriptional changes in nasal *versus* pulmonary airways *in vivo* has not been conducted, and little is known about the relative contribution of nasal *versus* pulmonary airways in health effects of upper airway irritants (Harkema et al., 1999; Bromberg, 2016). Such an assessment of upper *versus* lower airways would be critical in validating *in vitro* toxicity results derived from exposures of non-nasal respiratory cells (Cheah et al., 2013; Yeager et al., 2016; Xiong et al., 2018). Furthermore, given the neural and systemic endocrine effects of acrolein (Morris et al., 1999; Achanta and Jordt, 2017; Alewel et al., 2023b), a global transcriptional assessment of nasal and lung tissue could provide mechanistic insights into the potential contribution of circulating adrenal-derived stress hormones and local tissue changes in mediating nasal *versus* pulmonary response to acrolein.

Computational inhalation dosimetry approaches have been developed in rodents for inhalants such as acrolein to predict airway injury outcomes in humans (Cichocki et al., 2014). Owing to the primary deposition of acrolein in the nasal airways, greater injury responses in this region are expected; however, it is also likely that differential cellular composition and context with the surrounding milieu could influence the type of changes that occur in nasal *versus* lung tissue. Among the histologically unique zones of the rat nasal mucosa, the respiratory epithelium (used here for transcriptomics) comprises nearly 45% of the nasal surface area and experiences approximately 80% of airflow (Harkema, 1990; Chamanza and Wright, 2015). Further, this zone possesses cuboidal cells, which can metabolically interact with acrolein through GSH conjugation and other acrolein metabolizing enzymes, and brush cells, which play a role in nasal irritant detection (Saunders et al., 2013; Chamanza and Wright, 2015). Based on site-specific respiratory distribution of acrolein upon inhalation, we hypothesized that an assessment of global transcriptomic alterations in nasal *versus* pulmonary tissues would provide mechanistic insights regarding the pathways/gene signatures that are differentially impacted. More specifically, gene signatures elicited by acrolein may indicate patterns consistent with responses to adrenal-derived stress hormones involved in acrolein pathophysiology (Snow et al., 2017; Alewel et al., 2023b). In this study, we exposed male and female Wistar-Kyoto (WKY) rats nose-only to air or acrolein for an acute 4 h exposure duration and examined lavage markers of injury and inflammation. Due to exacerbated responses in males when compared to females in terms of nasal effects and propensity for neuroendocrine activation (Alewel et al., 2023b), we further performed nasal and pulmonary mRNA sequencing (RNAseq) in male rats to assess acrolein-induced changes in mRNA expression.

## 2 Materials and methods

### 2.1 Animals

Male and female WKY rats for both cohorts were purchased from Charles River Laboratories (Raleigh, NC). Animals were housed in the U.S. EPA Office of Research and Development Research Triangle Park animal facility accredited by the Association for Assessment and Accreditation of Laboratory Animal Care at atmospheric housing conditions of 21°C ± 1°C, 50%–65% relative humidity under a 12 h light/dark cycle. Rats were pair-housed in polycarbonate cages containing hardwood chip bedding and Enviro-dri wrinkle paper for enrichment, and provided with Purina (5,001) rat chow (Brentwood, MO) and water *ad libitum*, except during exposure. Exposure occurred at 12–13 weeks of age, and males weighed ∼250–300 g and females weighed ∼150–200 g. Sexes were housed and exposed separately. All experimental procedures received prior approval from the U.S. EPA Health Institutional Animal Care and Use Committee.

### 2.2 Experimental design

Data presented here for nasal and bronchoalveolar lavage injury markers were collected from our recent acrolein exposure study, cohort 1 (Alewel et al., 2023b). A new cohort of male rats (cohort 2), used for collecting tissues for transcriptomic analysis, underwent exact same exposure conditions. In brief, rats for each cohort were exposed to air or acrolein (*n* = 8/sex/group) (exposures for each sex conducted separately). Over 2 consecutive days prior to exposure, rats were acclimated to nose-only inhalation tubes (Lab Products, Seaford, DE). On day 1, rats underwent two 2 h acclimations 4 h apart, and on day 2, rats underwent a single 4 h acclimation. On the day of exposure, veterinary-grade artificial tears (Akorn Animal Health, Inc., Lake Forest, IL) were applied to the eyes and rats were exposed via nose-only inhalation to air (0 ppm) or acrolein for ∼4 h. Previously, to assess in real-time acrolein concentration-related effects on respiratory parameters during the 4 h exposure period, head-out plethysmography (HOP) was performed using a graded half-log exposure concentration paradigm, where for the first 30 min rats were exposed to 0, 0.10, 0.316, and 1 ppm acrolein (7.5 min/concentration) followed by 3.5 h of exposure at 3.16 ppm in the same animals. A separate air control group was also included that was exposed in parallel to filtered air for 4 h. Plethysmography, serum/plasma, and some nasal lavage fluid (NALF) and bronchoalveolar lavage fluid (BALF) data are reported in detail in our prior publication (Alewel et al., 2023b). To be consistent with that exposure paradigm, in this study for collecting nasal and lung tissue, the same protocol was used. Necropsies for sample collection in each cohort occurred immediately after exposure. Although the highest concentration of acrolein used here (3.16 ppm) is higher than ambient traffic-related acrolein pollution levels (De Woskin et al., 2003), woodsmoke and mainstream cigarette smoke contain up to 50 ppm and 90 ppm acrolein, respectively (Einhorn, 1975; Hristova et al., 2012).

### 2.3 Acrolein exposures

Acrolein was obtained as a gas stored under inert nitrogen for transport (Airgas, Morrisville, NC). For exposures, mass flow controllers modulated flow of acrolein and dilution air (purified air) to generate desired concentrations using a modified in-house gas blending system (MKS, Andover, MA). Acrolein was directed to flow through a 52-outlet port nose-only inhalation tower with air flow of 0.35 L/min per inhalation port. Targeted acrolein concentrations delivered to nose-only ports in exposure tower were continuously measured during exposure using an Agilent 6890 gas chromatography analyzer with flame ionization detection and 624 capillary columns (Supelco, Bellefonte, PA), and flows were adjusted as necessary to maintain the targeted acrolein concentrations (no deviations in target concentration were noted). Temperature (21.8°C ± 0.3°C) and relative humidity (38.9% ± 2.7%) in the room where the nose-only chambers were located were monitored once per hour during exposure.

### 2.4 Necropsy and sample collection

Immediately after exposure, rats were transported for necropsy to be weighed and euthanized via an intraperitoneal injection of sodium pentobarbital (>200 mg/kg) (Fatal-plus; Covetrus, Portland, ME) in a staggered manner between air- and acrolein-exposed rats (all necropsies completed within 1-2 h after end of exposure). For the animals in the first cohort, BALF was collected. The left lung lobe was tied off, and calcium- and magnesium-free phosphate buffered saline (PBS) warmed to 37°C was instilled into the lung through the trachea. BALF instillation volume was based upon a total lung capacity being 28 mL/kg body weight and right lung weight as approximately 60% of total lung weight. NALF was collected from the same cohort of animals through a retrograde instillation of 2 mL warm PBS through the pharyngeal opening of the nasal cavity, allowing lavage fluid to freely drip through nares. Another 1 mL of air was instilled in the same manner to collect any residual NALF. Aliquots of whole NALF and BALF were used for total cell quantification, using a Z1 Coulter Counter (Coulter, Inc., Miami, FL). Albumin levels in NALF and BALF were assessed using kits from Sekisui Diagnostics (Burlington, MA), with the protocol for this assay adapted for use on a Konelab Arena 30 clinical analyzer (Thermo Chemical Lab Systems, Espoo, Finland). From the second cohort of animals, un-lavaged left lung was removed and snap frozen in liquid nitrogen. From the same cohort, the head was removed and split down the sagittal crest to reveal the nasal cavities. Lateral and septal wall nasal respiratory epithelium was scraped from the nasal cavity, collected on parafilm, and frozen in liquid nitrogen. Olfactory epithelial tissues, which are visually distinct, were avoided while collecting septal and lateral wall respiratory epithelial tissues. In rodents, the olfactory mucosa is anatomically blocked from main inspired airflow (Harkema, 1990; Chamanza and Wright, 2015). Therefore, to not introduce variability in mRNA assessment that may arise from differential expression profiles between structurally and functionally unique nasal mucosal zones, we focused our assessment on nasal respiratory epithelium. All samples were stored at −80°C for later RNA analysis.

### 2.5 Cytokine quantification

NALF and BALF cytokines interleukin-6 (IL-6), tumor necrosis factor-α (TNF-α), C-X-C motif chemokine ligand 1 (CXCL1), interleukin-1β (IL-1β), interferon-γ (IFN-γ), and interleukin-13 (IL-13) were analyzed using V-PLEX pro-inflammatory panel 2 rat kits (Mesoscale Discovery Inc., Rockville, MD). The manufacturer’s protocol was followed for assessment in multiplex format. Electrochemiluminescence signals were measured using MESO QuickPlex SQ 120 platform (Mesoscale Discovery Inc., Rockville, MD). Protein signals were measured using a 4-parameter logistic model to determine sample concentration using MSD Discovery Workbench analysis software (Mesoscale Discovery Inc., Rockville, MD).

### 2.6 RNA isolation and mRNA sequencing

Nasal epithelial tissue (*n* = 5-6/group) and uniform portions of the left lung (*n* = 6–8/group) from male rats were used for RNA extraction and processed with RNeasy mini kits (Qiagen, Valencia, CA) as directed by the manufacturer. Following extraction, RNA quantity and purity (260/230 and 260/280 ratios) were determined using a Nanodrop 1000 (ThermoFisher Scientific Inc., Waltham, MA). RNA integrity was assessed by the RNA 6000 LabChip^®^ kit and a 2100 Bioanalyzer (Agilent Technologies, Santa Clara, CA) using a RIN cutoff of 7. mRNA samples were randomized and processed on an Apollo324 automated system (Takara Bio Inc., Kusatsu, Japan) for library prep with PrepX mRNA 48 protocol v19, using the PrepX^TM^ RNA-seq for Illumina Library Kit (Takara), SuperScript III reverse transcriptase (ThermoFisher), and AMPure XP Beads (Agilent). PCR amplification with 48 index primers was run for 16 cycles and resulting PCR product quality was analyzed again via Qubit (ThermoFisher) and Bioanalyzer (Agilent). An RNAseq library was prepared with Wafergen’s PrepX mRNA 48 protocol and dsDNA products were prepared from cDNA. Each library was sequenced according to Illumina NextSeq 500, with a final concentration of 2.2 pM + 2% PhiX and run for 75 cycles (Illumina Inc., San Diego, CA).

### 2.7 General statistical analysis

The data for NALF and BALF albumin, total cells, and cytokine proteins were analyzed using GraphPad Prism software v9. Due to low basal levels of proteins in NALF, some samples for albumin content in air-exposed groups were below the detection limit (BDL). For these samples, BDL values were imputed with the limit of detection (LOD) using LOD/√2. Likewise, in some cytokines, particularly among air-exposed controls in nasal lavage, few samples that were BDL were imputed in the same manner using LOD/√2 for each analyte for statistical comparison. First, outliers were identified and discarded using a robust regression outlier test (ROUT) with a false discovery threshold of 0.01 (Q = 1%). To meet the assumptions of normality and homoscedasticity within an analysis of variance (ANOVA), data underwent Shapiro-Wilks normality (α = 0.05) and Levene’s equality of variance tests. Data that did not meet ANOVA assumptions of equal variance and/or normality were Log transformed [Y = Log (Y)] prior to analysis. NALF and BALF markers were analyzed separately using a two-way ANOVA (sex, exposure). A Tukey’s multiple comparisons post-hoc was used and significance was determined at *p* ≤ .05.

### 2.8 Nextseq data normalization, statistics, and bioinformatics

Sequenced mRNA reads were mapped to the rat genome (rn7) using ensemble release 105 in the Partek Flow suite, R version 4.1.3. In total, 24,091 genes were mapped. Genes that did not have at least 1 read in 10 samples were removed, leaving 20,580 genes. An average of 15.5 million reads were mapped per sample with a standard deviation of 5.5 million. One nasal sample was removed because of low total reads (∼300,000). Principal components analysis was performed to identify any samples that did not cluster and should be removed; none were removed in this step. The remaining samples had 15.8 million reads per sample with a standard deviation of 5.3 million, with an average read per gene of 766. Differential gene expression was assessed in DESeq2 package in R (v1.34.0) (Love et al., 2014) and normalized reads were assessed for acrolein effects. Differential expression analyses were performed within tissue to assess the effects of acrolein. Gene expression was considered different when the fold change (FC) was greater than 1.2 (upregulation) or less than 0.833 (downregulation) and the Benjamini–Hochberg false discovery rate ≤ 0.1. Sequencing data is deposited in the National Center for Biotechnology Information Gene Expression Omnibus under accession number GSE247698.

For each heatmap, normalized read counts for each gene were averaged and calculated as the standard deviation from the mean, reflecting row z-score of each gene as up- (yellow) or downregulated (blue). Genes were clustered according to average-linkage Euclidean distancing using the online tool http://heatmapper.ca (last accessed 22 June 2023). We utilized Ingenuity Pathway Analysis (IPA) software (QIAGEN, Redwood City, CA; www.qiagen.com/ingenuity; last accessed 22 June 2023), which compares our mRNA expression dataset to previously reported databases and reports overlaps in expression changes for interpretation of major pathway and gene changes. Top canonical pathways were selected based upon significance between overlap of molecules in our dataset and molecular changes consistent with a particular network (adj. *p*-value <0.1). To infer involvement of upstream regulators, we determined an |activation z-score| ≥ 2 for meaningful prediction of network connectivity with over- or under-expressed transcripts (Krämer et al., 2014).

## 3 Results

### 3.1 Acrolein inhalation causes differential nasal versus pulmonary injury and inflammation

**FIGURE 1:**
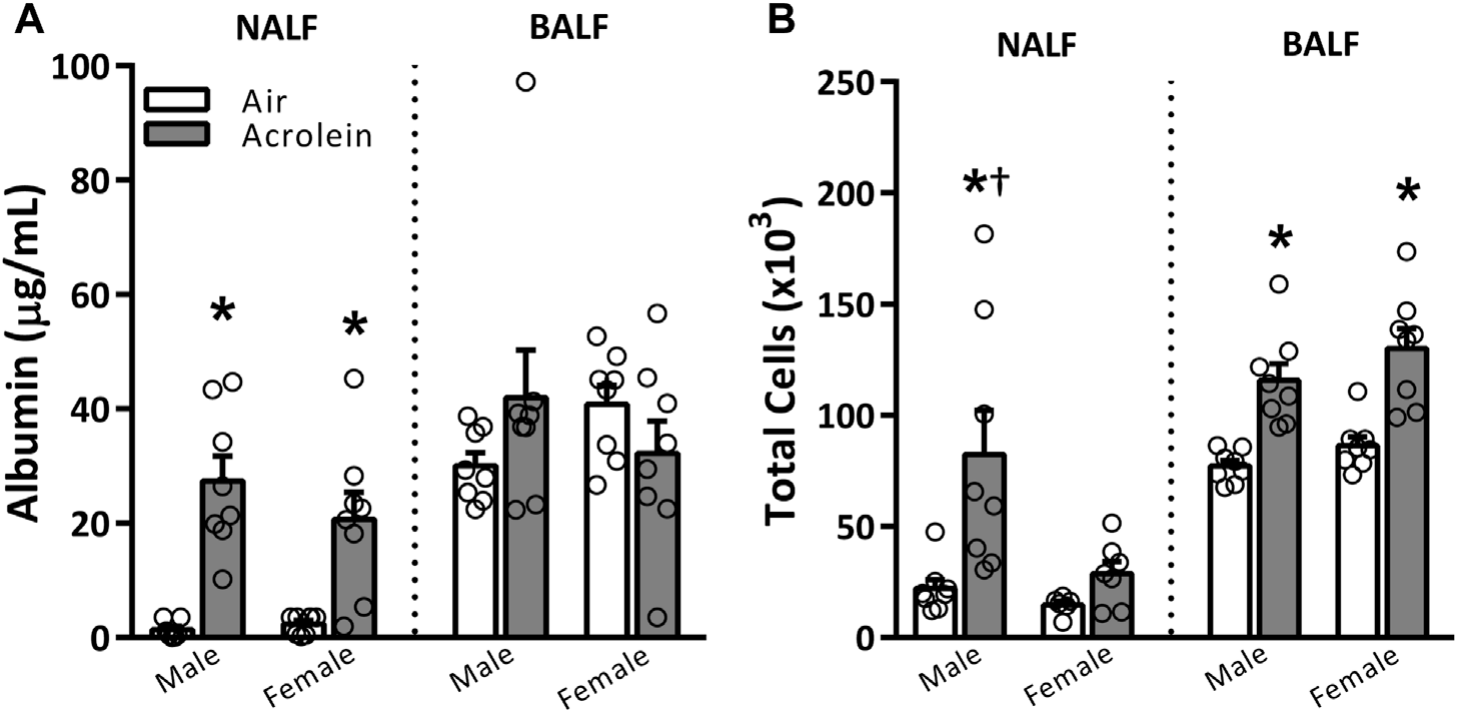
Acute acrolein inhalation induces both vascular leakage and inflammation in nasal airways but only inflammation in the pulmonary airways (males > females). Markers of injury and inflammation, albumin **(A)** and total cell counts **(B)**, respectively, were measured in the nasal lavage fluid (NALF) and bronchoalveolar lavage fluid (BALF) in samples collected immediately following air or acrolein inhalation. Data are mean ± SEM. * indicates a significant acrolein effect; † indicates a significant sex-effect (*n* = 6–8/group; *p* < .05).

To assess overall upper and lower airway injury and inflammation patterns in response to acrolein inhalation, we measured albumin and total cells in NALF and BALF (Figure 1). A single exposure to acrolein resulted in higher NALF albumin levels in male and female rats when compared to respective air groups, but did not change BALF albumin levels (Figure 1A). Interestingly, although both sexes demonstrated nasal protein leakage, only acrolein-exposed males had higher NALF total cell counts, which we previously reported as neutrophilic and eosinophilic inflammation (Alewel et al., 2023b) (Figure 1B). Further, acrolein exposure significantly elevated BALF total cell counts in both sexes (Figure 1B).

### 3.2 Acrolein induces a site- and sex-specific pro-inflammatory cytokine response

**FIGURE 2:**
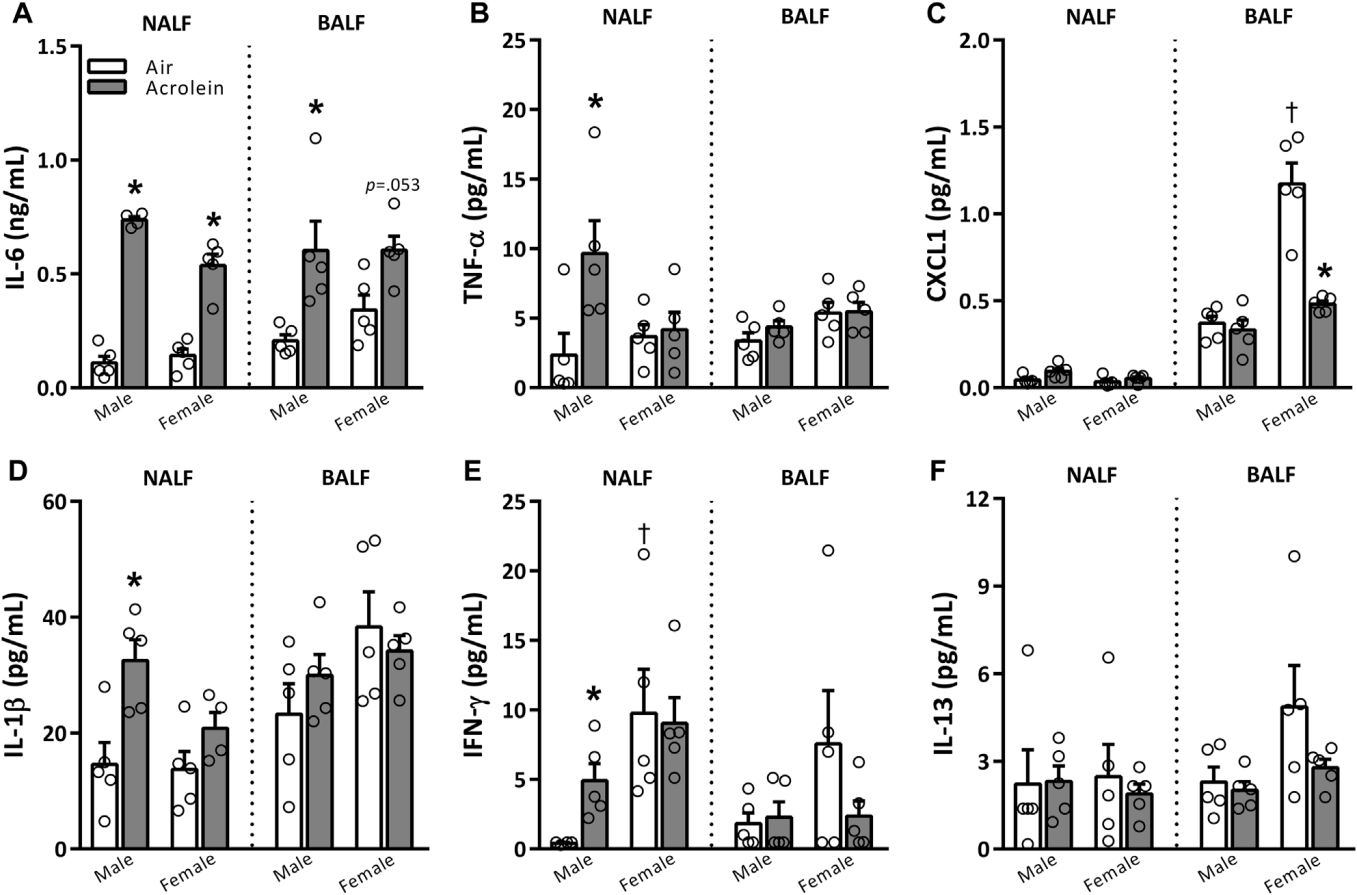
Acrolein alters respiratory pro-inflammatory cytokine levels in a sex- and site-specific manner. A panel of pro-inflammatory cytokine proteins were assessed in nasal lavage fluid (NALF) and bronchoalveolar lavage fluid (BALF), including interleukin-6 (IL-6, **(A)**), tumor necrosis factor-α (TNF-α, **(B)**), CXC motif ligand 1 (CXCL1, **(C)**), interleukin-1β (IL-1β, **(D)**), interferon-γ (IFN-γ, **(E)**), and interleukin-13 (IL-13, **(F)**). Data are mean ± SEM. * indicates a significant acrolein effect; † indicates a significant sex-effect (*n* = 4-5/group; *p* < .05).

NALF and BALF samples were further assessed to examine the potential involvement of various pro-inflammatory cytokines (Figure 2). The only cytokine impacted in both sexes was IL-6, which, compared to air group, was higher in NALF of acrolein-exposed males and females and BALF of acrolein-exposed males, and nearly significant in female BALF (*p* = 0.053) (Figure 2A). Other cytokine alterations followed a sex-specific response only in NALF, suggesting an effect at the site of nasal acrolein deposition. TNF-α, IL-1β, and IFN-γ levels were higher in NALF of acrolein-exposed males, whereas no other changes in these cytokines were noted in NALF of females or BALF of either sex (Figures 2B, D, E). CXCL1 and IL-13 NALF and BALF levels were not impacted by acrolein exposure in either sex, except for a decrease in BALF CXCL1 in acrolein-exposed females (Figures 2C, F). Few cytokines showed differences among air control groups, where NALF IFN-γ levels and BALF CXCL1 levels were higher in female air controls compared to males (Figures 2C, E).

### 3.3 Acrolein-induced mRNA expression changes differ between nasal and lung tissue

**FIGURE 3:**
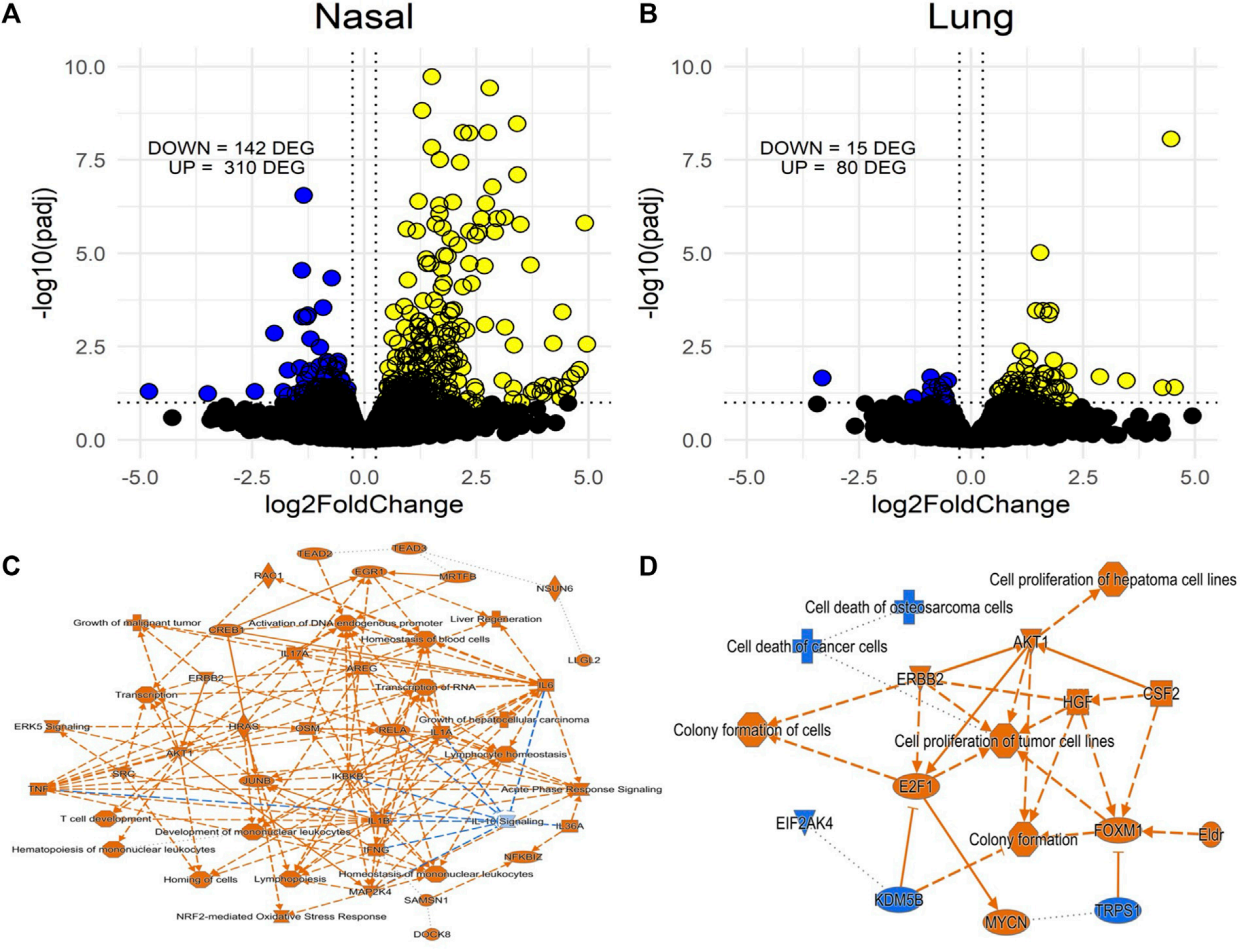
Volcano plots of differentially expressed genes (DEGs) from RNAseq in nasal **(A)** and lung tissue **(B)** of acrolein-exposed male rats and accompanying Ingenuity Pathway Analysis (IPA) activity plots showing primary signaling networks being overrepresented or underrepresented in nasal epithelial **(C)** and lung tissue **(D)**. More transcriptomic alterations were observed in nasal tissue, 310 upregulated and 142 downregulated DEGs, compared to lung tissue, which had 80 upregulated and 15 downregulated DEGs. Activity plots show overall inferred relationships between molecules and functions based on DEGs. Significance threshold was set at an absolute fold change in expression >20% (Log2FC) and adj. *p*-value <.1 (-Log10padj), with cutoffs shown by dotted lines on volcano plots (blue are downregulated and yellow are upregulated DEG). For activity plots, color indicates a positive (orange) or negative (blue) relationship, and lines indicate a direct (solid) or indirect (dashed) relationship.

To assess nasal *versus* lung transcriptional responses to acrolein, we performed global mRNA analysis in each tissue. Only males were used due to their greater susceptibility to acrolein-induced airway injury and inflammation when compared to females. Figure 3 shows volcano plots of RNAseq data obtained from nasal and lung tissues. Acrolein inhalation changed expression of 452 genes in the nasal epithelium (310 upregulated and 142 downregulated) (Figure 3A). Comparatively, acrolein exposure resulted in nearly 5-fold fewer DEGs in lung, changing expression of 95 genes (80 upregulated and 15 downregulated) (Figure 3B). To determine the overall effects on biological processes within nasal and lung tissue, IPA core analysis was performed. Figures 3C, D show the graphical summary of the interactive influence on key signaling regulators up- or downregulated after acrolein exposure in the nasal and lung tissue, respectively. This core analysis illustrates relations among predicted biological pathways, transcription factors, and important signaling modulators being impacted by acrolein exposure in the nasal *versus* lung tissue. Overall, tissue-specific differences can be seen in the number and function of biological processes involved in transcriptional alterations (Figure 3). This IPA core analysis demonstrates that cell signaling pathways and transcriptional activations involving innate inflammatory processes mediated by oxidative stress are induced in the nasal tissues with inhibition of IL-10 signaling. The increases in mRNA transcriptions for IL-6, IFN-γ, IL-1β, and TNF genes (Figure 3C) coincide with increases in these cytokine proteins in the NALF. The absence of these pathways activated in the lung is consistent with the lack of increases in these cytokines in the BALF. Rather, the pathways changed in the lung tissue show that cell growth and proliferation processes might be activated (Figure 3D).

### 3.4 Top DEGs and canonical pathways vary between nasal and lung tissue

**FIGURE 4:**
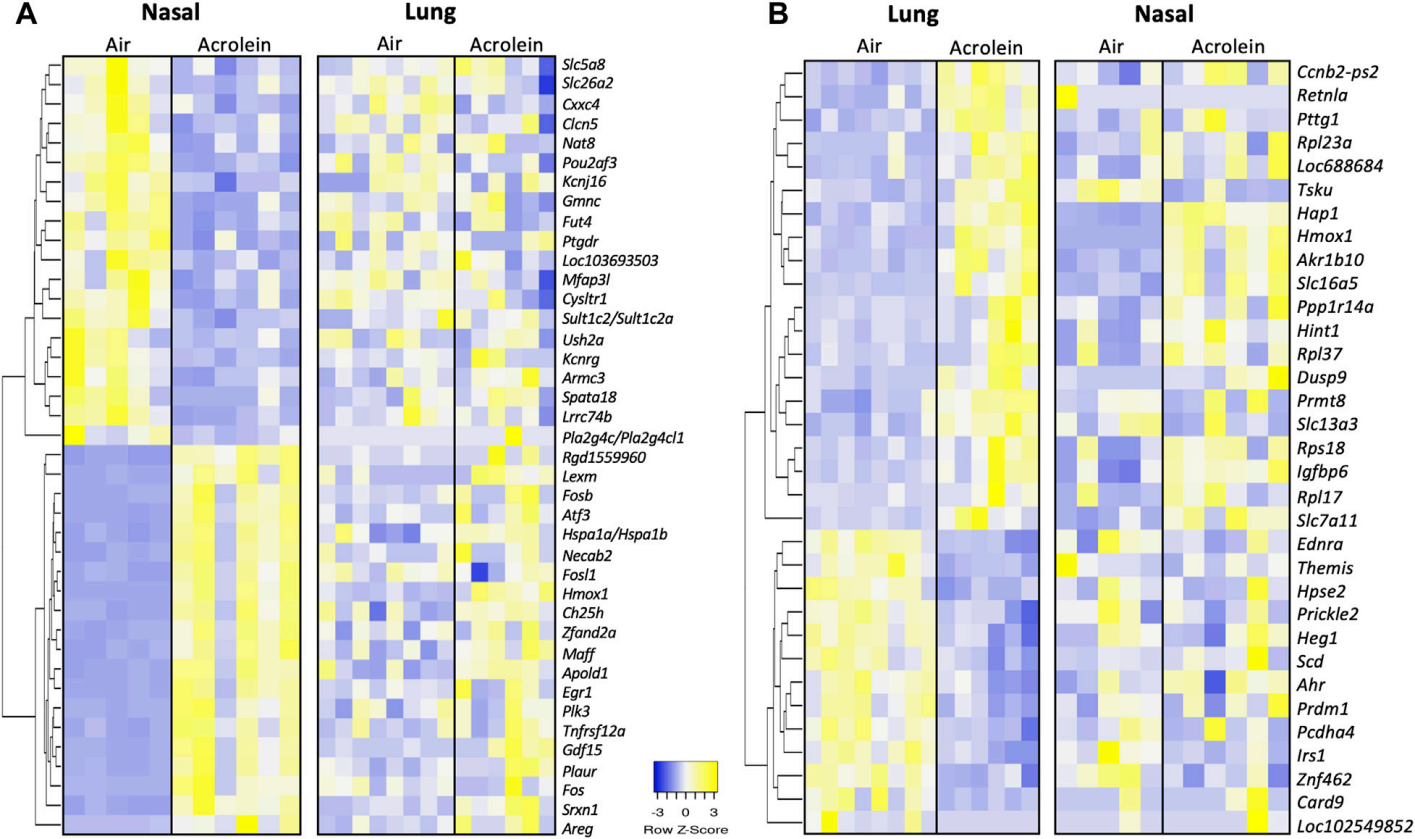
Heatmaps of top 20 up- and 20 downregulated differentially expressed genes (DEGs) in either nasal tissue with matching lung genes **(A)** or lung with matching nasal tissue genes **(B)**. Genes in either nasal tissue or lung were selected based upon highest or lowest fold change in expression, where row z-score are shown in heatmap hierarchically clustered by average-linkage Euclidean distance (nasal, *n* = 5-6; lung, *n* = 6–8). The same genes were selected and match ordered for tissue comparison of top DEGs. Upregulated genes are shown in yellow and downregulated genes are shown in blue. Supplementary Table S1 provides further gene information.

To highlight the similarities and differences of acrolein effects on cellular processes between the nasal and pulmonary tissues, the top 20 up- and 20 downregulated genes in nasal tissues were compared with same genes in the lung and *vice versa* (Figure 4). The heat map in Figure 4A displays the top 20 DEGs up- and downregulated in the nose (ranked based on fold change), with the listing of changes in same genes in the lung. In the nose, the most upregulated gene was *Hmox1* (Log2FC = 4.63), a gene regulating iron homeostasis and oxidative imbalance (Chen et al., 2020), and the most downregulated gene was *Gmnc* (Log2FC = −1.40), a gene involved in the differentiation of ciliated epithelial cells (Zhou et al., 2015) (Figure 4A). Other top upregulated nasal genes included multiple FOS family genes (*Fos*, *Fosl1*, and *Fosb*) involved in acrolein toxicity (Yeager et al., 2016)*,* and those downregulated included genes regulating ion channel functions and cellular transport (Figure 4A). It is noteworthy most of these same genes did not respond to acrolein exposure in lung tissue. While acrolein exposure caused only a fifth of the number of genes to be differentially expressed in the lung when compared to nasal airways, *Hmox1* was commonly upregulated in both tissues (Figure 4B). The heat map in Figure 4B displays the top DEGs in the lung from acrolein exposure, with the same genes selected and matched for nasal airways for comparison. In the lung, the most upregulated DEG was *Slc16a5* (Log2FC = 1.98) involved in transport of monocarboxylate metabolites such as pyruvate and lactate (Ren et al., 2022), and the most downregulated gene was *Card9* (Log2FC = −0.95), an upstream regulator involved in activation of NFκB and apoptosis signaling (Bertin et al., 2000). This pattern of change depicts that in the lung, acrolein transcriptional response likely did not involve the activation of NFκB signaling despite observed increases in BALF cells. Other genes downregulated in the lung included *Hpse2*, *Ahr*, and *Irs1*, all involved in cellular metabolism. Overall, few overlapping genes were differentially expressed in both tissues following acrolein exposure. We found 7 overlapping genes, *Hmox1*, *Cebpd*, *Akr1b10*, *Slc7a11*, *Slc16a5*, *Hap1*, and *Tsku*, differentially expressed in both nasal and lung tissue (Supplementary Table S2).

To obtain insight into processes affected by acrolein exposure, we performed canonical pathway analysis of mRNA expression data. Table 1 shows the top 15 most significantly altered pathways (adj. *p*-value <0.1) in each tissue following acrolein exposure. Notable pathways enriched by acrolein exposure in nasal tissue included acute phase response (APR) signaling, Nrf2-mediated oxidative stress, IL-6 signaling, unfolded protein response, ERK5 signaling, and HMGB1 signaling. Pathways downregulated by acrolein exposure in nasal RNAseq data were IL-10 and ATM signaling (Table 1). Unlike widespread transcriptional changes indicative of oxidative stress response and activation of inflammatory processes in the nasal tissue, the enriched canonical pathways in the lung indicated changes in xenobiotic metabolism.

**TABLE 1:** Canonical pathways identified by Ingenuity Pathway Analysis (IPA) in nasal and lung tissue suggests activation of oxidative stress and inflammatory pathways in nasal tissue and xenobiotic metabolism pathways in lung tissue. Table shows top directionally predicated canonical pathways in nasal and lung tissue (adj. *p* < 0.1). Canonical pathways shown are based upon *p*-value of overlap of differentially expressed genes (DEGs) with IPA networks, and include activation *z*-score, which predicts the activation state of a pathway (positive values in yellow indicates pathway activation, negative values in blue indicates pathway inhibition, and no highlight is non-directional). Pathway analysis in lung tissue yielded fewer associated pathways due to fewer transcriptional changes.

Canonical pathways	

**Nasal**	**adj. *p*-value**
	**(Activation z-score)**
IL-10 Signaling	7.24E-09
	(-0.5)
Acute Phase Response Signaling	9.77E-08
	(3.05)
NRF2-mediated Oxidative Stress Response	1.15E-07
	(2.50)
IL-17A Signaling in Fibroblasts	1.82E-07
	(0)
Role of JAK family kinases in IL-6-type Cytokine Signaling	7.94E-07
	(2.53)
Role of Macrophages, Fibroblasts and Endothelial Cells in Rheumatoid Arthritis	9.55E-07
	(0)
Wound Healing Signaling Pathway	1.35E-06
	(3.15)
Unfolded protein response	2.69E-06
	(2.65)
IL-17 Signaling	3.47E-06
	(3.74)
ERK5 Signaling	3.98E-06
	(3)
HMGB1 Signaling	5.13E-06
	(3.16)
ATM Signaling	6.92E-06
	(-0.45)
Macrophage Alternative Activation Signaling Pathway	8.51E-06
	(1.07)
IL-6 Signaling	1.15E-05
	(2.71)
IL-12 Signaling and Production in Macrophages	1.15E-05
	(0.26)
**Lung**	**adj. *p*-value**
	**(Activation z-score)**
Xenobiotic Metabolism AHR Signaling Pathway	9.77E-06
	(1.34)
Kinetochore Metaphase Signaling Pathway	3.24E-05
	(1.34)
Xenobiotic Metabolism Signaling	4.68E-05
	(0)
Xenobiotic Metabolism General Signaling Pathway	1.07E-04
	(1.34)
NRF2-mediated Oxidative Stress Response	1.29E-04
	(0)
Aryl Hydrocarbon Receptor Signaling	1.78E-04
	(0)
Xenobiotic Metabolism PXR Signaling Pathway	4.27E-04
	(2.24)

### 3.5 Altered acute phase response and Nrf2-mediated oxidative signaling is congruent with injury and inflammation markers in NALF and BALF

**FIGURE 5:**
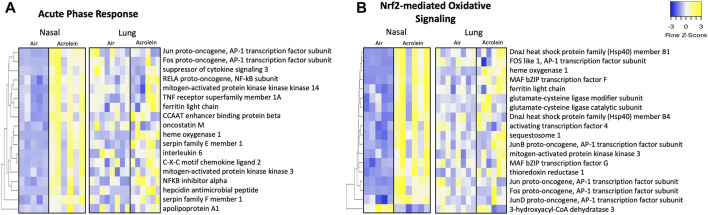
Acute phase response and Nrf2-mediated oxidative inflammatory signaling pathways are enriched in nasal but not lung tissue following acute acrolein inhalation. Inflammatory pathways of acute phase response **(A)** and Nrf2-mediated oxidative signaling **(B)** were among top enriched transcriptional pathways in nasal tissue. Nasal tissue mRNA heatmaps show differentially expressed genes (DEGs) within each pathway hierarchically clustered by average-linkage Euclidean distance. The same genes were selected from lung RNAseq results and ordered to match nasal clustering for tissue comparison of inflammatory and oxidative stress changes. DEGs were determined at an absolute fold change >20% and adj. *p*-value <.1 (nasal, *n* = 5–6; lung, *n* = 6–8). Yellow signifies increased gene expression and blue indicates decreased gene expression. Supplementary Table S3 shows Log2FC and adjusted *p*-value for each gene.

Figure 5A shows 18 significant DEGs within the APR network in nasal tissue. Multiple genes associated with APR activation were enriched in nasal but not lung tissue, including *Il6*, *Cxcl2*, *Hamp*, *Tnfrsf1a*, and *Serpine1*, corroborating NALF and BALF pro-inflammatory cytokine involvement (Figure 5A). Genes *Map2k3* and *Map3k14*, components of mitogen-activated protein kinase (MAPK) cascades in innate immune activation, were significantly enriched in nasal epithelium (Figure 5A). Acrolein’s effect on APR genes in the lung was smaller than that in the nasal tissue. Further, 18 DEGs comprised Nrf2-mediated oxidative signaling in the nose (Figure 5B). Various AP-1 subunit genes (*Fos*, *Fosl1*, *Jun*, *Junb*, and *Jund*) were enriched in the nose, along with *Gclc* and *Gclm* (Figure 5B). Among DEGs identified within these nasal pathways, only *Hmox1* was significantly altered in the lung (Figures 5A, B).

### 3.6 Glucocorticoid receptor and GPCR signaling is upregulated in nasal tissue of acrolein-exposed rats

**FIGURE 6:**
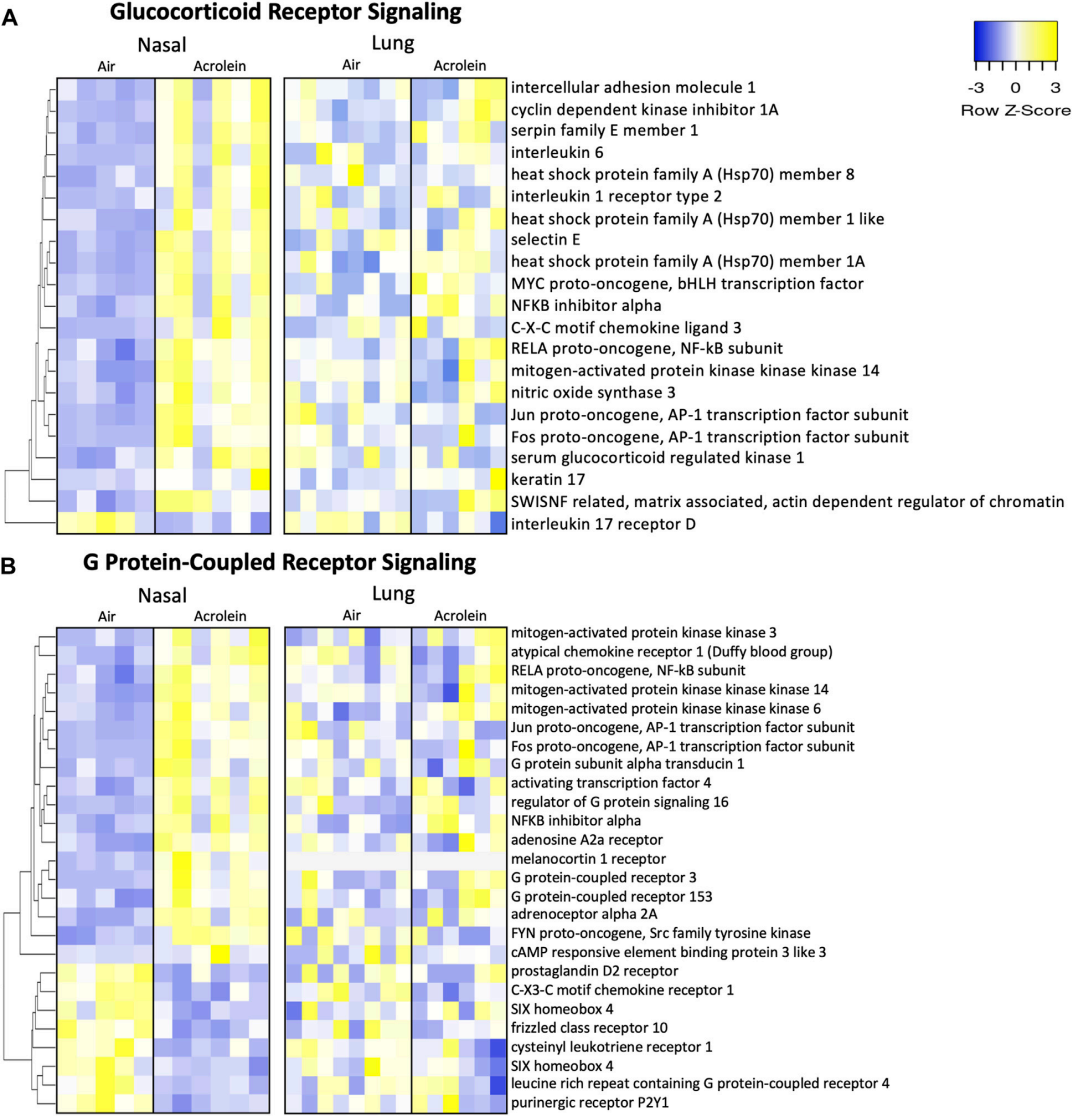
Acrolein-exposure alters glucocorticoid receptor (GR) and G protein-coupled receptor (GPCR) genes reflective of stress signaling in nasal epithelial but not lung tissue. Differentially expressed genes (DEGs) involved in GR signaling **(A)** and GPCR signaling **(B)** were identified in nasal tissue (*n* = 5-6) and hierarchically clustered by average-linkage Euclidean distance. These same genes were identified in lung (*n* = 6–8) and organized in the same manner for site-specific comparison-associated transcriptional alterations. Yellow indicates increased expression and blue indicates decreased expression (fold change >20%; adj. *p*-value <.1). Supplementary Table S4 shows Log2FC and adj. *p*-value for each gene.

To explore the involvement of nuclear receptor signaling in upper and lower airway acrolein effects, we identified molecules pertaining to glucocorticoid receptor (GR) and G protein-coupled receptor (GPCR) signaling through IPA analysis (Figure 6), which is potentially affected based on our prior observation that circulating corticosterone and epinephrine are increased in rats after acrolein exposure (Snow et al., 2017; Alewel et al., 2023b). Overall, acrolein-exposed rats exhibited many gene expression changes modulated by GR and GPCR signaling in the nose that were not observed in the lung. Prominent genes enriched in the nasal GR signaling pathway included Hsp70 encoding genes (*Hspa1a/Hspa1b*, *Hspa1l*, and *Hspa8*) and *Sgk1* (Figure 6A), which is a glucocorticoid-regulated kinase prompted by cellular stress (Jang et al., 2022). Multiple GPCR signaling network changes were observed in the nose following acrolein exposure, whereas these effects were not seen in the lung (Figure 6B). GPCR-mediated changes altering intracellular adenylate cyclase or cyclic-AMP (cAMP) levels were noted through increased nasal expression of genes such as *Adra2a*, *Adora2a*, *Creb3l3*, and *Atf4*. Among the 21 GR and 26 GPCR signaling mRNA expression changes noted in the nasal tissue, none of these genes were significantly changed in the lung (Figure 6).

Because one of the objectives of our transcriptomic analysis was to identify the involvement of adrenal-hormone signaling in acrolein airways effects, we evaluated specific glucocorticoid-responsive genes that are extensively validated as consistent markers of GR activity due to their gene loci often falling within glucocorticoid response elements (GRE) (Wang et al., 2004; Kan and Himes, 2021). Table 2 shows 9 such genes selected from nasal and lung RNAseq data. *Ccl2*, *Cebpd*, *Per1*, *Sgk1*, *Thbd*, *Ccl20*, and *Gadd45b* were significantly enriched in nasal tissue only, except for *Cebpd* which shared increased lung expression. Interestingly, *Fkbp5*, a prominent modulator of glucocorticoid signaling, was enriched only in the lung, also seen in prior ozone studies (Henriquez et al., 2021).

**TABLE 2:** Well characterized glucocorticoid-responsive genes are changed in nasal and lung tissue following acrolein exposure. Genes validated by prior ‘omics studies as consistently regulated by glucocorticoid-mediated cellular signaling are shown by fold change (FC) in nasal (*n* = 5-6) and lung tissue (*n* = 6–8), where a FC in bold with an * represents a significant change in expression following acrolein exposure (adj *p*-value <0.1). Supplementary Table S5 displays adj. *p*-value for each gene.

*Gene Symbol*	Gene name	Nasal	Lung
***Ccl2***	chemokine (C-C motif) ligand 2	4.15*	0.98
***Cebpd***	CCAAT enhancer binding protein delta	3.67*	1.91*
***Per1***	period circadian regulator 1	2.02*	1.33
***Sgk1***	serum/glucocorticoid regulated kinase 1	1.73*	0.89
***Thbd***	thrombomodulin	1.66*	0.82
***Tsc22d3***	TSC22 domain family, member 3	1.42	1.16
***Fkbp5***	FKBP prolyl isomerase 5	1.14	1.76*
***Ccl20***	C-C motif chemokine ligand 20	2.47*	1.22
***Gadd45b***	growth arrest and DNA damage inducible beta	2.14*	1.56

### 3.7 Upstream predictor analysis of acrolein-induced mRNA expression changes

We used the IPA upstream predictor tool to evaluate cytokines, transcriptional activators, or chemicals which shared similar gene changes with acrolein-induced airway effects (Table 3). Here, our gene expression dataset was compared to IPA databases to provide the most likely regulators responsible for network changes in either nasal or lung datasets. Nasal acrolein-induced changes predicted activated regulation by lipopolysaccharide (LPS), multiple cytokines, CREB1, and inhibition by various chemical and biologic agents (Table 3). Further, ozone and cigarette smoke were predicted chemical toxicants with gene signatures similar to acrolein-induced nasal effects, which is consistent with acrolein as a major tobacco smoke constituent (Hikisz and Jacenik, 2023), (Supplementary Table S6). Forskolin and methylprednisolone were also suggested upstream predictors of nasal transcriptomic changes (Supplementary Table S6), implicating the involvement of epinephrine-mediated GPCR signaling via activation of cAMP and corticosteroid effects on GR in acrolein-induced upper airway effects. The fewer transcriptional changes in the lung, however, were predicted to be related to different substances. The lung prediction algorithm identified similarities to activation of aflatoxin B1, NFE2L2 (Nrf2), and Vegf, as well as inhibition by multiple transcriptional regulators and chemical agents (Table 3).

**TABLE 3:** Unbiased Ingenuity upstream predictor analysis of top predicted activators and inhibitors in nasal and lung tissue. Top 10 activated or inhibited upstream regulators in nasal and lung tissue associated with acrolein-induced transcriptional alterations are shown. IPA-identified upstream regulators based upon network associations with acrolein-induced transcriptional alterations in nasal or lung tissue. Activation z-score denotes regulator activation (>0) or inhibition (<0), where the magnitude of direction is depicted by higher absolute values. Supplementary Table S6 shows molecules falling within each regulator dataset, as well as additional regulators.

Tissue	Upstream regulator	Molecule type	Predicted activation state	Activation z-score	*p*-value of overlap
**Nasal**	lipopolysaccharide	chemical drug	Activated	7.46	1.40E-28
	tetradecanoylphorbol acetate	chemical drug		6.79	1.53E-31
	TNF	cytokine		6.65	3.91E-37
	IL1B	cytokine		6.42	1.17E-36
	CREB1	transcription regulator		6.15	5.61E-23
	NFkB (complex)	complex		6.09	5.41E-23
	IFNG	cytokine		5.99	5.08E-26
	PDGF BB	complex		5.83	3.60E-32
	poly rI:rC-RNA	biologic drug		5.82	2.37E-18
	AGT	growth factor		5.66	1.66E-17
	PD98059	chemical - kinase inhibitor	Inhibited	−5.71	1.23E-27
	LY294002	chemical drug		−5.44	4.26E-29
	U0126	chemical drug		−5.02	1.40E-30
	SB203580	chemical drug		−4.91	7.59E-20
	SP600125	chemical drug		−4.87	6.38E-28
	NC410	biologic drug		−4.11	6.17E-13
	THZ1	chemical drug		−3.99	3.44E-10
	bisindolylmaleimide I	chemical drug		−3.96	5.95E-11
	Alpha catenin	group		−3.94	1.45E-11
	MSB0011359C	biologic drug		−3.87	6.78E-13
Lung	aflatoxin B1	chemical	Activated	4.00	9.96E-16
	Eldr	other		3.16	2.06E-15
	CSF2	cytokine		2.99	9.42E-07
	HGF	growth factor		2.90	6.89E-08
	NFE2L2	transcription regulator		2.86	1.23E-07
	1,2-dithiol-3-thione	chemical reagent		2.80	3.22E-07
	beta-estradiol	chemical - endogenous mammalian		2.68	1.65E-09
	PTGER2	G-protein coupled receptor		2.65	6.18E-08
	decitabine	chemical drug		2.61	4.07E-04
	Vegf	group		2.56	6.32E-06
	KDM5B	transcription regulator	Inhibited	−2.45	7.21E-06
	GSR	enzyme		−2.24	9.59E-08
	TRPS1	transcription regulator		−2.24	5.76E-07
	LARP1	translation regulator		−2.24	6.98E-06
	l-asparaginase	biologic drug		−2.24	3.35E-05
	TXNRD1	enzyme		−2.21	2.71E-07
	let-7	microRNA		−2.18	9.47E-07
	fulvestrant	chemical drug		−2.16	3.39E-06
	methylprednisolone	chemical drug		−2.03	4.21E-03
	EIF2AK4	kinase		−2.00	2.57E-05

## 4 Discussion

Volatile water-soluble vapors, such as acrolein, deposit in nasal airways of both rats and humans, but a greater proportion may deposit into nasal airways in rats than the tracheobronchial space in humans because of differences in airway morphology, cellular composition, breathing mode, and ventilation rate (Corley et al., 2012). Although computational approaches have been developed to predict airway injury in humans based on rodent inhalation dosimetry data (Cichocki et al., 2014), there remains considerable uncertainty in predicting human toxicity because sex- and site-specific variation in respiratory responses are not well defined. In this study, we assessed nasal (isolated nasal respiratory epithelial tissue) *versus* pulmonary responses to acrolein inhalation in rats to aid in understanding the molecular signatures of cellular effects in these compartments and how systemically released stress hormones might be modulating local nasal *versus* pulmonary injury and inflammation. Consistent with our previous findings (Alewel et al., 2023b), acute acrolein inhalation caused marked nasal injury and cytokine changes, with males being more sensitive than females. Transcriptomic analysis of nasal respiratory epithelium *versus* lung tissue of male rats revealed major differences in terms of the extent and type of changes following acrolein inhalation. There were 452 genes differentially expressed in acrolein-exposed nasal tissue *versus* only 95 genes in the lung. The changes in the nose were reflective of the activation of innate immune responses, characterized by NFκB, TNF, oxidative stress, Nrf2 signaling, and glucocorticoid and GPCR signaling. The lung showed fewer mRNA expression changes that were indicative of changes in xenobiotic metabolism and proliferation. These divergent responses may reflect the acrolein concentration encountered at the site of airway deposition, with likely localized influence of circulating stress hormones, and/or variance in site-specific cellular biology (including cell type) in the nose and lung.

Acute airway effects of aldehydes involve the induction of pro-inflammatory cytokine networks (van der Toorn et al., 2013; Sun et al., 2014; Xiong et al., 2018). Given that IL-6 was the only acute phase reactant increased in nasal tissue of both males and females, and in the BALF of males, this suggests a distinct mechanism that is not influenced by sex in the acrolein response that may serve as a generalized, sensitive indicator of inflammation. Higher TNF-α, IL-1β, and IFN-γ in acrolein-exposed male but not female NALF suggest sex-specific influence on these innate immune cytokines, perhaps through complex hormonal interactions. Glucocorticoids and steroidal hormones, including androgens/estrogens, are known to interactively regulate immune responses (Bereshchenko et al., 2018), which may underlie greater sensitivity of male rats to acrolein-induced inflammation under these conditions. Considering this exposure scenario has been shown to increase glucocorticoids only in males (Alewel et al., 2023b), further studies are needed to elucidate possible interactions between adrenal and gonadal hormones in sex-specific outcomes. Since acrolein induced nasal eosinophilic and neutrophilic extravasation in male rats (Snow et al., 2017; Alewel et al., 2023b), the cytokine results suggest a site-specific inflammatory response, whereby TNF-α, IFN-γ, and IL-1β play a critical signaling role in the nasal airways (Takeuchi and Akira, 2010; Aghasafari et al., 2019). Lastly, the lack of BALF cytokine changes despite increased inflammatory cells may indicate complex homeostatic responses to acrolein, whereby redistribution of circulating immune cells through adrenal-derived stress hormones (Dhabhar et al., 2012; Henriquez et al., 2017; Snow et al., 2017; Henriquez et al., 2018) might occur through the entire respiratory system under stress.

Previously reported inflammatory and oxidative stress-related gene expression and signaling in the nasal and tracheobronchial mucosa (Cichocki et al., 2014; Moghe et al., 2015) align well with transcriptional changes we observed in the nasal tissue after an acute acrolein exposure. In contrast to the few changes that were observed in the lung transcriptome in the present study, marked transcriptomic changes have been reported in the lungs of susceptible mouse strains after an acute acrolein inhalation, albeit at 3-4 times higher concentrations than what we used in the present study (Fabisiak et al., 2011). Further, C57BL/6J mice exposed to lethal concentrations of acrolein (50 ppm) showed pulmonary transcriptomic changes of inflammatory and oxidative cascades similar to current nasal results (Bein et al., 2021), suggesting the muted lung responses in the present study is related to limited lung deposition. Despite significantly lower oxidative and inflammatory changes in the lung, the pathway analysis of transcriptional data revealed activation of cell growth and proliferation-mediated processes by a long non-coding downstream RNA (Eldr) involved in cancer cell growth and promotion (Sur and Ray, 2022). It is likely that acrolein metabolites, which might have been encountered by the lung, may activate genes involved in systemically encountered small non-coding RNA-induced effects (Tang et al., 2011).

Top DEGs in the nose were most reflective of previously established molecular effects of acrolein toxicity, including the activation of Fos-Jun transcriptional machinery, as well as enhanced *Atf3* that can cause ER stress and activation of cellular survival pathways (Chinenov and Kerppola, 2001; Haberzettl et al., 2009; Moghe et al., 2015). In the lung, aryl hydrocarbon receptor (AHR) and pregnane X receptor (PXR) xenobiotic signaling pathways were enriched, which are nuclear receptor pathways that coordinate xenobiotic metabolizing enzymes and cellular detoxification processes in response to environmental chemical stimuli (Oladimeji and Chen, 2018; Lamorte et al., 2021). Additionally, because of shared marked increased expression of *Hmox1* and *Slc7a11*, a pair of genes that work in tandem to modulate iron homeostasis and cysteine/glutamate transport for cellular GSH biosynthesis (Poss and Tonegawa, 1997; Thompson and Burcham, 2008; Tang et al., 2021), it appears that acrolein-induced respiratory effects likely involve disrupted iron homeostasis processes in the nose and distal lung space.

Similar to ozone, acute acrolein inhalation increases circulating epinephrine and corticosterone (Snow et al., 2017; Alewel et al., 2023b). Ligand-activated GR and GPCR are two of the most transcriptionally active nuclear signaling receptors across the genome and are highly responsive to environmental stressors (Alhosaini et al., 2021; Frigo et al., 2021). Although adrenergic receptors (AR) and GR likely play a key role in mediating pulmonary health effects of various pollutants (Hodge et al., 2021), their involvement in nasal effects of acrolein is not established. The factors involved in acrolein stress, such as AP-1 and NFκB, are often co-regulated with GRE (Reddy et al., 2009). In this study, we noted GR and GPCR signaling pathways were enriched in the nasal tissue that included gene targets such as *Hsp70*, a co-chaperone essential for nuclear trafficking of GR (Pratt et al., 2006). Even though BALF analysis revealed no lung injury, glucocorticoid-responsive *Fkbp5*, encoding scaffolding proteins for GR assembly to modulate cellular sensitivity to glucocorticoids (Hartmann et al., 2021; Nold et al., 2021), was enriched only in the lung. In prior studies, we have shown the involvement of *Fkbp5* in coordinating pulmonary and hypothalamic changes following exposure to ozone and particulate matter (Henriquez et al., 2019; Alewel et al., 2023a). Nasal mRNA expression was enriched for *Adra2a*, as well as the adenylate cyclase gene *Adora2a*, which both interact with the PI3K/AKT signaling pathway responsive to catecholamines (Shi et al., 2019; Ye et al., 2023). Genes modulated through catecholamine-induced cAMP intracellular messaging, such as *Creb3l3*, *Atf4*, and *Ptgdr*, were also enriched in the nose, perhaps linked with the activated cAMP-dependent transcription factors such as CREB1 predicted to be involved in upstream predictor analysis. Overall, these changes suggest that while GR and AR signaling mechanisms were activated in the nasal tissue, the lung also displayed some degree of glucocorticoid involvement in response to acrolein, but in a manner that was different than the nose.

Multiple nasal upstream regulators were predicted, as expected, to be similar to the effects of cigarette smoke, glucocorticoids, and inflammatory signaling regulators of oxidative stress. Chemicals or drugs that target molecular modes of acrolein toxicity, such as AP-1 and PI3K/AKT activity (Wan et al., 2022; Wu et al., 2022), were predicted as inhibited in IPA analysis, suggesting anti-inflammatory responses in the nose were inhibited. Interestingly, IPA predicted nasal mRNA effects significantly overlapped with prior ozone datasets, indicating airway effects of these volatile gases share similar yet site-specific oxidative and inflammatory responses. No top regulators were found to overlap between the nose and lung, although with low transcriptional alterations in the lung, there is a lower confidence in network prediction of upstream regulators. Nevertheless, multiple transcriptional regulators identified in acrolein-induced lung mRNA changes control cell cycle checkpoint and proliferation processes (Dey et al., 2008; Sun et al., 2012; Wu et al., 2014), suggesting upstream proto-oncogene effects in the lung that could have implications for dysregulated cellular growth over time.

It is important to highlight the limitations of this study, particularly as they pertain to the implications of these findings. The hypothesis generating observations in this study will need to be confirmed with targeted interventional approaches in both sexes. This nasal and lung transcriptional assessment did not involve long-term effects or temporality examination, as the changes observed immediately post exposure are likely reversible. A repeated or chronic acrolein exposure could trigger divergent mechanisms involved in inflammatory and stress signaling processes (Moghe et al., 2015), meaning respiratory toxicity outcomes of rodent or *in vitro* experiments should be analyzed in the context of the model being employed. Future investigations would benefit from assessing the potential habituation or desensitization of these nasal responses to intermittent or repeated acrolein exposure.

Here, we demonstrate that a single acrolein inhalation exposure produced nasal injury and inflammation while causing only a small inflammatory response in the lung, with the effects being more pronounced in male than female rats. Transcriptional responses in nasal *versus* lung tissue of acrolein-exposed male rats revealed quantitative and qualitative differences between tissues owing to expected depositional differences. Distinct transcriptional changes in the nasal tissue were characterized by the activation of major oxidative/antioxidative pathways, proinflammatory signaling, acute phase response, ER stress, glucocorticoid and GPCR signaling, and inhibition of anti-inflammatory mechanisms, whereas cellular proliferative responses and changes in xenobiotic metabolic processes were observed in the lung. Although pulmonary airways are likely not exposed to the same concentration of acrolein as nasal tissue, based on the distinct type of changes observed, it can be surmised that the biological processes that are affected between nasal airway *versus* pulmonary tissues, while influenced by similar circulating factors such as stress hormones and metabolites, are mechanistically different. Considering acute acrolein exposures are frequently encountered through wildfires, tobacco smoking, and anthropogenic pollution, these findings may help elucidate the pathophysiology of upper and lower airway disease affecting large segments of the population.

## Data Availability

Publicly available datasets were analyzed in this study. This data can be found here: https://www.ncbi.nlm.nih.gov/geo/query/acc.cgi?acc=GSE247698.
